# Assessment and Standardization of Follow-Up Notes Among Healthcare Providers in the Internal Medicine Unit at Mek Nimir Hospital: A Two-Cycle Clinical Audit From February to December 2025

**DOI:** 10.7759/cureus.115595

**Published:** 2026-09-01

**Authors:** Mohamed Osman Elhag Osman, Abdelhameed Awad, Mohamed F Mohamed, Awad Osama Kamal Ibrahim, Ahmed Mahmoud Sidahmed Abdullah, Ammar Osman, Mohamed Alfatih E Omer

**Affiliations:** 1 Internal Medicine, Federal Ministry of Health, Khartoum, SDN; 2 Internal Medicine, Omdurman Teaching Hospital, Omdurman, SDN; 3 Orthopaedic Surgery, Burjeel Hospital, Abu Dhabi, ARE; 4 Medicine and Surgery, Elrazi University, Khartoum, SDN; 5 Pulmonary Medicine, Aswan University Hospitals, Aswan, EGY; 6 General Practice, Al Mamoon Medical Center, Al Ain, ARE; 7 Emergency Department, Omdurman Teaching Hospital, Omdurman, SDN

**Keywords:** bsoap, clinical audit, clinical documentation, documentation quality, follow-up notes, internal medicine, patient safety, sudan

## Abstract

Complete follow-up notes support continuity of care by communicating the patient's current symptoms, objective findings, clinical assessment, and management plan. This clinical audit evaluated whether a standardized Background, Subjective, Objective, Assessment, and Plan (BSOAP) format could improve the completeness of inpatient follow-up documentation in the Internal Medicine Unit at Mek Nimir Hospital.

A prospective before-and-after, two-cycle clinical audit was conducted using unpaired groups of inpatient follow-up notes. The baseline cycle reviewed 42 notes from February 10 to October 1, 2025. After introduction of a standardized BSOAP format, focused staff teaching, and feedback on documentation gaps, the re-audit reviewed 14 notes from December 1 to December 25, 2025. Each note was assessed for the five BSOAP domains and for complete documentation of all five domains. Two-sided Fisher's exact tests were used to compare proportions because the cycle samples were unpaired, unequal in size, and included small or zero cell counts.

Subjective documentation increased from 0/42 (0.0%) at baseline to 14/14 (100.0%) at re-audit, Assessment documentation increased from 7/42 (16.7%) to 13/14 (92.9%), and complete BSOAP documentation increased from 0/42 (0.0%) to 11/14 (78.6%); all three changes were statistically significant (p<0.001). Objective and Plan documentation remained at 100.0% in both cycles. Background documentation decreased from 42/42 (100.0%) to 12/14 (85.7%), but the difference was not statistically significant (p=0.059).

A structured BSOAP follow-up format supported by focused teaching and audit feedback was associated with substantial improvement in documentation completeness. Continued use of the format and repeated audit cycles are needed to sustain the improvement and address the remaining gaps in Background and Assessment documentation.

## Introduction

Clinical audit is a quality-improvement process in which current practice is compared with explicit standards, deficiencies are identified, changes are introduced, and performance is measured again to determine whether improvement has occurred [1]. In inpatient medicine, follow-up documentation is a central part of that process because it records the patient's current clinical status, active problems, and management plan for the next healthcare provider.

Medical records serve several functions, but their primary purpose is to support patient care. Structured record keeping has also been associated with benefits for patient outcomes and clinicians' performance [2]. In busy clinical environments, inconsistent or incomplete notes can create omissions in the clinical narrative and weaken continuity between different doctors, shifts, and levels of seniority. Structured tools and checklists can help reduce omissions and improve consistency in complex, high-workload settings [3].

The Background, Subjective, Objective, Assessment, and Plan (BSOAP) framework extends the familiar Subjective, Objective, Assessment, and Plan (SOAP) structure by adding a Background domain. Recent clinical audits in Sudan have reported improved progress-note documentation after introducing structured BSOAP formats together with education, visual reminders, feedback, or supervision [4,5]. These findings provide a relevant local rationale for evaluating a similar approach in an internal medicine setting.

Before the intervention, follow-up documentation in the unit did not use a standardized BSOAP format, and the baseline audit identified important gaps, particularly in Subjective and Assessment documentation. BSOAP was selected because it retains the familiar SOAP structure while adding a Background component that provides relevant clinical context, including the admission diagnosis, relevant history, comorbidities, medications, allergies, and admission circumstances. This additional context may be particularly useful during serial inpatient reviews involving multiple healthcare providers.

For this audit, Background summarized relevant history, diagnosis, comorbidities, current medications, allergies, and admission context. Subjective recorded the patient's current complaint, symptom progression, or a statement that there was no new complaint. Objective included vital signs, examination findings, investigations, imaging, and monitoring data. Assessment recorded the clinician's interpretation, diagnosis, or active problem list. Plan recorded treatment, monitoring, investigations, referrals, and follow-up actions.

The aim was to improve and standardize follow-up notes among doctors in the Internal Medicine Unit at Mek Nimir Hospital between February and December 2025. The objectives were to improve the completeness and consistency of follow-up documentation and provide a clearer structure that could support communication and continuity of care between healthcare providers. Patient-safety outcomes were not directly measured.

## Technical report

Materials and methods

Study Design and Setting

This was a prospective before-and-after, two-cycle clinical audit conducted in the Internal Medicine Unit at Mek Nimir Hospital, Sudan. The baseline cycle assessed follow-up documentation before implementation of the standardized BSOAP intervention, and the re-audit assessed documentation after implementation. 

Different patient batches were reviewed during the two audit cycles; therefore, the note samples were unpaired. However, the same group of healthcare providers contributed documentation during both cycles, and potential provider-level clustering was not accounted for in the note-level analysis.

Study Population and Sampling

The audit population consisted of inpatient follow-up notes written by healthcare providers involved in adult internal medicine care, including house officers and other doctors contributing to daily follow-up documentation. Available notes from the defined audit periods were reviewed. A total of 56 follow-up notes were included: 42 in the baseline cycle and 14 in the re-audit. The difference in sample size reflected the different durations of the predefined audit periods and the number of eligible follow-up notes available during each period. Equal sample sizes were not prespecified.

Available inpatient follow-up notes from the predefined audit periods were reviewed. Inclusion criteria were routine follow-up notes for adult patients admitted under the Internal Medicine Unit, written by doctors involved in inpatient care, and documented within the relevant audit period. Notes from other departments or outside the predefined audit periods were not included.

Audit Periods

The baseline cycle was conducted from February 10 to October 1, 2025. The re-audit was conducted from December 1 to December 25, 2025 after implementation of the documentation intervention.

Audit Standard and Criteria

For this local audit, the target standard was 100% completion of each BSOAP domain. A follow-up note was considered fully complete only when all five domains were documented in the same note. The operational scoring definitions are shown in Table 1. Two members of the clinical audit team independently assessed the follow-up notes using the predefined BSOAP criteria. Disagreements or ambiguous entries were resolved through discussion and consensus before assigning the final classification. Formal inter-rater agreement was not calculated.

**Table 1:** BSOAP documentation criteria and local audit standard. BSOAP: Background, Subjective, Objective, Assessment, and Plan; GCS: Glasgow Coma Scale; RBG: random blood glucose; UOP: urine output.

Domain	Documentation requirement used for scoring	Audit standard
Background (B)	Relevant history, known diagnosis, comorbidities, medications, allergies, admission context, or previous clinical history.	1
Subjective (S)	Current complaint, new complaint, symptom progression, patient-reported concern, or statement of no new complaint.	1
Objective (O)	Vital signs, physical examination, GCS, oxygen saturation, RBG, UOP, investigation results, imaging, or other measurable clinical findings.	1
Assessment (A)	Diagnosis, working diagnosis, clinical impression, active problem list, or interpretation of clinical status.	1
Plan (P)	Medications, monitoring, investigations, procedures, supportive care, specialty plan, follow-up, or continuation of management.	1
Complete BSOAP	All five domains documented in the same follow-up note.	1

Baseline Cycle and Intervention

The baseline cycle evaluated 42 follow-up notes. Objective and Plan components were consistently recorded, whereas Subjective and Assessment components were frequently absent or not clearly documented. Following review of the baseline findings, the unit introduced a standardized BSOAP follow-up format, brief teaching sessions on the five domains, and feedback on common documentation gaps. Before the re-audit, five brief teaching sessions were delivered by the clinical audit team to doctors involved in inpatient follow-up documentation. The sessions reviewed the five BSOAP domains, demonstrated practical use of the standardized format, and addressed common gaps identified during the baseline audit, particularly Subjective and Assessment documentation. Doctors were encouraged to record symptom progression or a statement of no new complaint, provide an explicit clinical assessment, and document a clear management plan. The intervention combined a structured tool with targeted education and feedback, an approach consistent with implementation literature emphasizing context-specific, multifaceted strategies for changing clinical practice [6].

Re-audit

The re-audit evaluated 14 follow-up notes after implementation of the intervention. The same BSOAP criteria and completion definitions used during the baseline cycle were applied.

Data Analysis

Each BSOAP domain was coded as present or absent. Frequencies and percentages were calculated for each domain in both cycles. Complete BSOAP documentation was coded as present only when all five domains appeared in the same note. Absolute percentage-point change was calculated by subtracting the baseline percentage from the re-audit percentage. Two-sided 95% confidence intervals for differences in proportions were calculated using the Newcombe method based on Wilson score intervals. Fisher’s exact test was used to compare independent proportions because the cycles were unpaired, the sample sizes were unequal, and several contingency-table cells contained small or zero counts. Statistical significance was defined as p<0.05. Data management, descriptive analysis, and statistical calculations were performed using Microsoft Excel, Version 2607 (Build 20228.20158; Microsoft Corporation, Redmond, WA, USA), included with Microsoft Office Professional Plus 2024.

Ethical Considerations

The audit evaluated routinely generated inpatient documentation for local quality-improvement purposes. Patient identifiers were excluded from the submitted audit material.

Results

A total of 56 follow-up notes were analyzed across the two audit cycles. The baseline cycle included 42 notes and the re-audit included 14 notes. Descriptively, the mean BSOAP completeness score increased from 3.17/5 (SD 0.38) at baseline to 4.79/5 (SD 0.43) at re-audit. This composite score was reported descriptively, and no inferential statistical comparison was performed. At baseline, 35 notes scored three out of five and seven notes scored four out of five; no note contained all five domains. At re-audit, 11 notes scored five out of five and three notes scored four out of five.

The largest changes occurred in Subjective documentation, Assessment documentation, and complete BSOAP documentation. Subjective documentation increased from 0/42 (0.0%) to 14/14 (100.0%), Assessment documentation increased from 7/42 (16.7%) to 13/14 (92.9%), and complete BSOAP documentation increased from 0/42 (0.0%) to 11/14 (78.6%). All three changes were statistically significant (p<0.001).

Objective and Plan documentation were present in every reviewed note in both cycles and therefore showed no measurable change. Background documentation decreased from 42/42 (100.0%) to 12/14 (85.7%); this difference was not statistically significant (p=0.059). Comparative results are presented in Table 2.

**Table 2:** Documentation completeness before and after the BSOAP intervention. N (%) indicates the number and percentage of follow-up notes meeting each criterion. P-values were calculated using two-sided Fisher's exact tests for the unpaired note samples. Percentage-point change was calculated as the re-audit percentage minus the baseline percentage. Values in parentheses accompanying percentage-point changes represent 95% confidence intervals calculated using the Newcombe-Wilson method. P-values were calculated using two-sided Fisher's exact tests. Statistical significance was defined as p<0.05. BSOAP: Background, Subjective, Objective, Assessment, and Plan

Criterion	Baseline n (%)	Re-audit n (%)	Change, percentage points (95% CI)	p-value
Background	42 (100.0%)	12 (85.7%)	-14.3 (-39.9 to -1.0)	0.059
Subjective	0 (0.0%)	14 (100.0%)	+100.0 (+76.9 to +100.0)	<0.001
Objective	42 (100.0%)	14 (100.0%)	0.0 (-21.5 to +8.4)	1
Assessment	7 (16.7%)	13 (92.9%)	+76.2 (+48.2 to +86.4)	<0.001
Plan	42 (100.0%)	14 (100.0%)	0.0 (-21.5 to +8.4)	1
Complete BSOAP	0 (0.0%)	11 (78.6%)	+78.6 (+51.1 to +92.4)	<0.001

## Discussion

This two-cycle clinical audit found a marked improvement in the completeness and standardization of follow-up documentation after introduction of a structured BSOAP format with staff teaching and audit feedback. The greatest gains occurred in Subjective documentation, Assessment documentation, and completion of all five domains within the same follow-up note.

The baseline findings suggest that clinicians were already consistently recording measurable clinical information and management plans, as Objective and Plan documentation were complete in every reviewed note. The main gaps were the absence of a clear patient-reported update and the low rate of explicit clinical assessment. These omissions can make a progress note harder to interpret because the relationship between the patient's current symptoms, the clinician's interpretation, and the management plan is less clear.

The observed improvement is consistent with other Sudanese clinical audits. Hamed et al. reported improvement across BSOAP components after combining a structured framework with local teaching, visual reminders, discussion, and supervision in a pediatric setting [4]. Idres et al. similarly reported that overall adherence to BSOAP documentation increased from 31.1% in the first cycle to 84.9% in the second cycle after staff training and introduction of a structured template [5]. Together with the present audit, these studies suggest that structured documentation interventions are feasible in Sudanese hospital settings and can improve documentation completeness.

The intervention used in this audit was deliberately simple: a standard structure, brief education, and feedback on observed gaps. This is compatible with broader implementation evidence showing that changes in clinical practice often require strategies tailored to the local setting rather than dissemination of a standard alone [6]. Structured tools can also reduce reliance on memory and help prevent omission of expected items in demanding clinical environments [3].

Complete BSOAP documentation improved from no complete notes at baseline to 78.6% at re-audit. This is a clinically relevant improvement in documentation quality, but it should not be interpreted as evidence of improved patient outcomes because this audit measured the presence of documentation domains rather than clinical outcomes, diagnostic accuracy, or treatment appropriateness. The results support better documentation structure and continuity of information, not a direct causal effect on morbidity, mortality, or length of stay.

Background documentation decreased from 100.0% at baseline to 85.7% at re-audit; this observed decrease did not meet the prespecified threshold for statistical significance (p=0.059). Future versions of the template should therefore retain a mandatory Background field for the admission diagnosis, important comorbidities, relevant medications, allergies, and other context needed to understand the current review.

Healthcare provider satisfaction with the BSOAP format was not formally assessed in this audit. A future cycle could include a brief pre-intervention and post-intervention survey assessing clarity, ease of use, perceived communication quality, and documentation burden.

Limitations

This audit was conducted in a single internal medicine unit, which limits generalizability to other departments and hospitals. The two cycles used different patient batches and unequal sample sizes, so the analysis was unpaired. The re-audit sample was smaller than the baseline sample, which reduces precision. The same group of healthcare providers contributed follow-up documentation during both audit cycles, and individual providers may have contributed multiple notes. Consequently, potential provider-level clustering was not accounted for in the note-level statistical analysis. The audit assessed the presence or absence of documentation elements rather than the clinical accuracy or appropriateness of the information recorded.

Recommendations and sustainability

The next audit cycle should focus on sustaining the improvements in Subjective documentation while closing the remaining gaps in Background and Assessment documentation. Repeated feedback and periodic re-audit should be used to determine whether performance remains stable over time. The recommended actions for sustaining and extending these improvements are summarized in Table 3.

**Table 3:** Recommended action plan for sustaining and extending improvement. BSOAP: Background, Subjective, Objective, Assessment, and Plan

Issue identified	Recommended action	Responsible person	Timeline	Re-audit measure
Background omitted in some re-audit notes	Make Background a mandatory field covering diagnosis, comorbidities, medications, allergies, and admission context.	Internal medicine audit lead and ward doctors	Within two weeks	Background documented in at least 95% of notes
Assessment not yet complete in every note	Add a required line for clinical impression or active problem list during each follow-up.	House officers and supervising doctors	Immediate	Assessment documented in at least 95% of notes
Need to sustain Subjective documentation	Continue documenting symptom progression, persistent symptoms, no new complaint, or new symptoms in every follow-up.	All doctors writing follow-up notes	Immediate	Subjective documented in at least 95% of notes
Provider satisfaction not measured	Introduce a short anonymous staff survey before and after template use.	Audit team	Next audit cycle	At least 70% staff response rate
Need ongoing monitoring	Repeat the BSOAP audit and present findings at departmental meetings.	Clinical governance lead	Every one to three months	Complete BSOAP documented in at least 90% of notes

## Conclusions

This two-cycle clinical audit demonstrated that a structured BSOAP follow-up format, supported by focused teaching and audit feedback, was associated with substantial improvement in the completeness of inpatient follow-up documentation in the Internal Medicine Unit at Mek Nimir Hospital. The strongest improvements were seen in Subjective and Assessment documentation and in completion of all five documentation domains within the same note.

Continued use of the standardized format, reinforcement of the Background and Assessment fields, and repeated audit cycles are warranted to determine whether the improvement can be sustained. Future work should also measure healthcare provider experience and evaluate whether better documentation is associated with improved handover quality or other patient-safety outcomes.

These findings should be interpreted in the context of a single-center audit and the relatively small re-audit sample of 14 follow-up notes.
